# Ecological Validity of Walking Capacity Tests in Multiple Sclerosis

**DOI:** 10.1371/journal.pone.0123822

**Published:** 2015-04-16

**Authors:** J. P. Stellmann, A. Neuhaus, N. Götze, S. Briken, C. Lederer, M. Schimpl, C. Heesen, M. Daumer

**Affiliations:** 1 Institute of Neuroimmunology and MS (INIMS) and Department of Neurology, University Medical Center Hamburg-Eppendorf, Hamburg, Germany; 2 Sylvia Lawry Centre for Multiple Sclerosis Research, Munich, Germany; 3 Trium Analysis Online GmbH, Munich, Germany; Medical University of Innsbruck, AUSTRIA

## Abstract

**Background:**

Ecological validity implicates in how far clinical assessments refer to real life. Short clinical gait tests up to ten meters and 2- or 6-Minutes Walking Tests (2MWT/6MWT) are used as performance-based outcomes in Multiple Sclerosis (MS) studies and considered as moderately associated with real life mobility.

**Objective:**

To investigate the ecological validity of 10 Meter Walking Test (10mWT), 2MWT and 6MWT.

**Methods:**

Persons with MS performed 10mWT, 6MWT including 2MWT and 7 recorded days by accelerometry. Ecological validity was assumed if walking tests represented a typical walking sequence in real-life and correlations with accelerometry parameters were strong.

**Results:**

In this cohort (n=28, medians: age=45, EDSS=3.2, disease duration=9 years), uninterrupted walking of 2 or 6 minutes occurred not frequent in real life (2.61 and 0.35 sequences/day). 10mWT correlated only with slow walking speed quantiles in real life. 2MWT and 6MWT correlated moderately with most real life walking parameters.

**Conclusion:**

Clinical gait tests over a few meters have a poor ecological validity while validity is moderate for 2MWT and 6MWT. Mobile accelerometry offers the opportunity to control and improve the ecological validity of MS mobility outcomes.

## Introduction

Persons with Multiple Sclerosis (pwMS) rate mobility as one of their most important bodily functions.[1] Multiple Sclerosis (MS) affects mobility in about three out of four patients and mobility impairment increases with disease duration.[2,3] The accepted standard of disability measurement in MS, the Extended Disability Status Scale (EDSS) refers to walking ability in the range between 20 and 500 meters.[4] Mobility restriction in MS is known to be associated with annual productivity loss, annual caregiver time and patients quality of life.[5] Physical inactivity might as well explain a higher risk for cardiovascular diseases in MS.[6] Beside MS, several studies have proven an association between mobility and morbidity, such as risk for dementia and cardiovascular diseases.[7] While the importance of mobility is widely accepted, the ideal measurement approach is still under discussion.[8,9]

Up to now, mobility assessment in MS is based mainly on short clinical gait test, i.e. performance tests as the Timed 25 foot walk (T25FW), the 10 m Walking Test (10mWT) and the 2- respectively 6-Minute Walking Test (2MWT/6MWT).[8,10] The use of performance tests is already restricted as they show high day-to-day variability and are influenced by the time of day.[11,12] Further on, it is unknown, how often 2 or 6 minutes of uninterrupted walking occur in real life and if changes in the performance of walking tests are associated with a change in real life mobility of pwMS.[13] However, treatment approvals already rely on these tests and the T25FW as well as the 6MWT have been recommended as patient relevant mobility outcomes.[9,10,13–15] Mobile accelerometry offers the possibility to gain ecologic valid, objective and reliable mobility data from pwMS in their daily life environment.[16–20] Over a period of 7 days, which might be most representative for peoples’ habits, it allows the assessment of gait speed and other parameters as distance, steps or sequences of a defined walking period. Gijbels et al. found a moderate association between clinical gait tests and the amount of steps within 7 days.[21] Motl et al. reported good correlations between total daily movement counts on the vertical axis and standard measurements as EDSS, T25FW, 6MWT and the self-reported walking scale MSWS-12.[22] Overall, short walking tests have shown only limited ecological validity.

To the best of our knowledge, it has not been investigated in depth, if short clinical tests might be ecologic valid for single aspects of real life mobility. We were interested how often per day pwMS perform uninterrupted walking sequences comparable to the clinical gait tests (e.g. 2 or 6 minutes) and if standard tests might represent specific walking speeds e.g. if a 10 m walk might represents short and faster walks in real life.

## Methods

The study was designed to investigate the length and frequencies of uninterrupted walking sequences during a 7-day frame. In a second step, cross-sectional correlations between gait tests and accelerometry data should clarify which aspects of real-life mobility might be represented by the clinical walking tests. We performed a data-driven explorative analysis of clinical gait tests and real-life walking speed parameters without any adjustment for multiple testing or a validation strategy with a second cohort.

### Patients

30 consecutive pwMS with an Expanded Disability Status Scale (EDSS) below 7 from the MS outpatient clinic of the UMC Hamburg were recruited for an explorative comparison of 10mWT, 2MWT and 6MWT with mobile accelerometry.[4,23] All participants gave written informed consent and the regional ethical review board (medical association Hamburg “Ethik-Kommission der Ärztekammer Hamburg”, study-ID: PV4405) approved the investigation. Patients were asked to perform the 10mWT and the 6MWT together with clinical scoring (EDSS) and wear the actibelt during the measurement and afterwards for another 7 days. EDSS rating was performed according to the guidelines from neurostatus.net by neurologists with an experience in MS. For the 10mWT patients were asked to walk ‘at fastest but safe speed’ from a static start.[10] Start and stop time was measured with a stopwatch by a single trained research assistant. For the 6MWT, patients were instructed ‘to walk at fastest speed, and to cover as much distance as possible’ according to published guidelines.[24] Subjects performed the test on a 20 m corridor with turning around at each end. After each minute passed, patients were informed about the time left but not encouraged.[10] As described by Gijbels et al. we defined the distance covered after 2 minutes of the 6MWT as distance for the 2MWT distance.[10] All participants were instructed how to use the actibelt. A trained instructor switched the actibelt on, to start the measurement over the next 7 days. Patients were asked to wear the actibelt except from showering, swimming or while sleeping. At the end of the 7 days period, patients switched off the actibelt and sent it back by mail. Two patients were excluded due to corrupted actibelt data preventing a meaningful analysis.

### actibelt

The actibelt is a tri-axial accelerometer with 100Hz sampling frequency; it has 512 megabytes of memory corresponding to 10 days continuous recording and a battery life of 20 days. The accelerometer is placed inside a belt buckle, which the wearer fixes around the waist by an elasticated belt. With this design, the device is discreet and unobtrusive, is located close to the subject’s centre of mass and on the sagittal symmetry plane of the body. It can either be used for long- term monitoring in a free-living environment (‘‘week-in-a-box”) or activity assessment in a clinical setting (‘‘rapid tests”).[18,25]

### Data analysis

We performed descriptive statistics of demographic variables for the cohort. We calculated mean walking speeds for the 10mWT and 6MWT (distance/time measured by the investigator).[26,27] Beside accelerometry adherence parameters (time of measurement, time of adherence, proportion of “up-side-down” time) we extracted the frequencies of uninterrupted walking sequences of 6 minutes, 2 minutes, 30 seconds and 15 seconds within one week. Standard gait parameters were extracted from the raw actibelt week measurement: distance per day, number of steps per day and hour, walking speed (mean = 50%-quantile, max, min) and walking speed in sequences with 50 or 100 consecutive steps. We assumed, that different clinical gait tests might be associated with different tasks in real-life mobility. Those tasks might be represented by different walking speeds, e.g. fast walking speeds to reach a ringing phone or slower speeds while taking a longer walk. We extracted for each patient a spectrum of walking speeds in quantiles of 5% steps. 0% indicated the lowest measurable individual walking speed, 50% the mean walking speed and 100% the highest individual measurable walking speed. Real-life walking speeds were calculated based on the most frequent uninterrupted walking sequences. Quantiles of walking speeds could only be calculated if at least ten walking sequences were available. We used the investigator measured 10mWT and 6MWT for analysis.[25,26]

We performed univariate linear regression analyses for the extracted actibelt data with 10mWT walking speed, 2MWT and 6MWT independently and calculated coefficient of determination R² and Pearson correlation coefficient as measures of association between the two included variables. Data were analysed using R software for statistical computing and graphics.[28]

## Results

### Summary data

The 28 patients of the cohort represented a typical moderately disabled MS population (median EDSS 3.2). Participants were predominantly female (64%), had a median age of 45 and were mainly in a relapsing-remitting MS (RRMS) disease course. The complete descriptive statistics of the cohort is presented in Table 1. The adherence to the 7-days accelerometry was very good with a median measurement time per day of 11.1 hours (range 6.5, 20.3).

**Table 1:** Descriptive statistics of the cohort.

	Cohort (n = 28)
Age	45 [27, 68] (38, 51)
Females n (%)	18 (64%)
Disease course n (%)	
RRMS	14 (50%)
SPMS	8 (28.5%)
PPMS	5 (18.9%)
Disease duration	9 [1, 24] (4, 17)
EDSS	3.2 [1, 6.5] (2.5, 4.1)
**Accelerometry**	
Measurement days per patient	7 [4, 9] (7, 8)
Average daily adherence hours per patient	11.1 [6.5, 20.3] (9.7, 13.1)

Data as median, range in square brackets, lower/upper quartiles in round brackets, RRMS = Relapsing-remitting MS, SPMS = secondary progressive MS, PPMS = primary progressive MS, 6MWT = 6 Minute Walking Test.

Descriptive statistics of the accelerometry measurement and the clinical gait tests are presented in Table 2. 10mWT walking speed (1.4 m/s) was higher than in the other tests (2MWT: 1.3 m/s; 6MWT: 1.4 m/s).

**Table 2:** Descriptive Statistics Outcomes.

Walking Tests (mean walking speeds)	
10mWT [m/s] n = 28	1.42 [0.4, 2.72] (1.15, 1.74)
2MWT [m/s] n = 23	1.31 [0.3, 1.75] (1.08, 1.58)
6MWT [m/s] n = 23	1.39 [0.45, 1.73] (1.11, 1.61)
**actibelt**	
Sequences of uninterrupted walking (n/day)	
6 minutes	0.35 [0, 2.88] (0.14, 0.58)
2 minutes	2.61 [0, 6.8] (1.1, 3.9)
30 seconds	11.3 [0, 19.9] (4.7, 14.9)
15 seconds	22 [0, 50] (12.5, 31.6)
Mean quantiles of walking speeds* [m/s]	
95%	1.57 [1.25, 2.01] (1.41, 1.63)
75%	1.41 [1.16, 1.73] (1.32, 1.47)
50%	1.29 [1.11, 1.6] (1.22, 1.36)
25%	1.19 [1.04, 1.39] (1.11, 1.23)
5%	1 [0.87, 1.12] (0.95, 1.06)
Distance per day (m)	4054 [1662, 6235] (3229, 5518)
Number of steps per day	5669 [304, 9254] (3894, 7625)
Uninterrupted walking sequence with at least 50 steps	
Walking speed [ms]	1.14 [0.97, 1.30] (1.1, 1.18)
Uninterrupted walking sequence with at least 100 steps	
Walking speed [m/s]	1.16 [1.05, 1.33] (1.12, 1.24)

Data as median, range in square brackets, lower/upper quartiles in round brackets*. Range not as whole numbers, as calculated numbers represent mean sequences per day in the one week measurement per patient.

*Walking speeds are based on sequences of at least 15 seconds of uninterrupted walking, quantiles of walking speeds refer to the range of individual walking speeds with 0% the lowest measurable and 100% the fastest measurable walking speed, 10mWT = 10 meter Walking Test, 6MWT = 6 Minute Walking Test.

**Fig 1:**
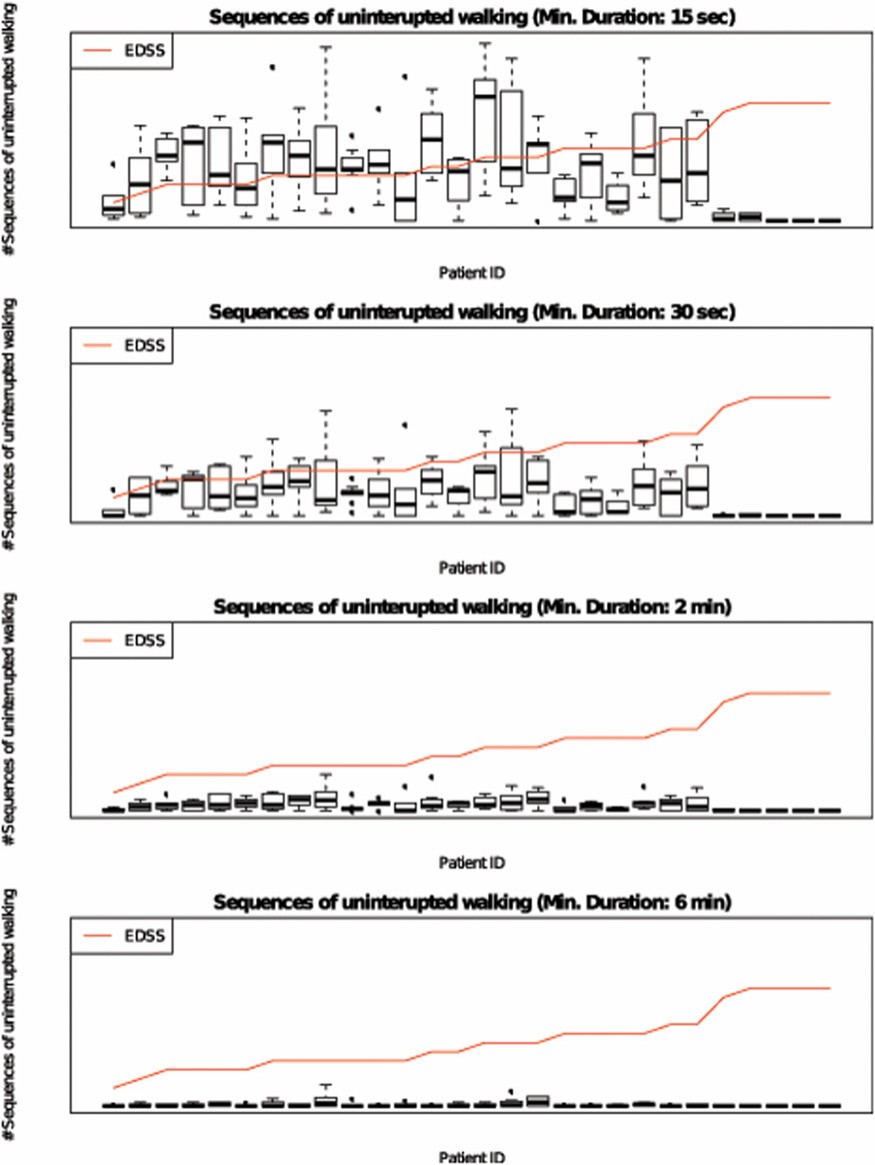
Sequences of 15s / 30s / 2 min / 6 min uninterrupted walking in real life. Mean number of uninterrupted walking sequences per day for each patient during a 7-day assessment of real life mobility with a mobile accelerometer. Data presented as boxplots with median, 25%-/75%-quantiles and whiskers representing 5%-/95%-quantiles.

6-minutes sequences of uninterrupted walking occurred only occasionally in real life (median 0.35 sequences per day). Even sequences of 2 minutes were registered less than 10 times a day (median 2.61 sequences per day) and sequences of 30 seconds 11.3 times per day. (Fig 1) For calculations of real-life walking speed we choose sequences of 15 seconds with a median of 22 sequences per day. Median walking speeds were 1 m/s (range 0.87–1.12) at the 5%-quantile of walking speed, 1.29 m/s (range 1.11–1.60) at the mean-50%-quantile and 1.57 (range 1.25, 2.01) at the 95%-quantile.

Two Patients from the cohort denied completing the 6MWT and another 3 actibelt measurements had to be excluded from correlation analyses as the number of walking sequences was to low to calculate reliable walking speeds. These five patients had an EDSS of 6 or above indicating the need of a walking assistance or maximal walking range of less than 100m. Therefore only patients with an EDSS below 6.0 (n = 23) were available for correlation analyses.

### 10mWT

**Fig 2:**
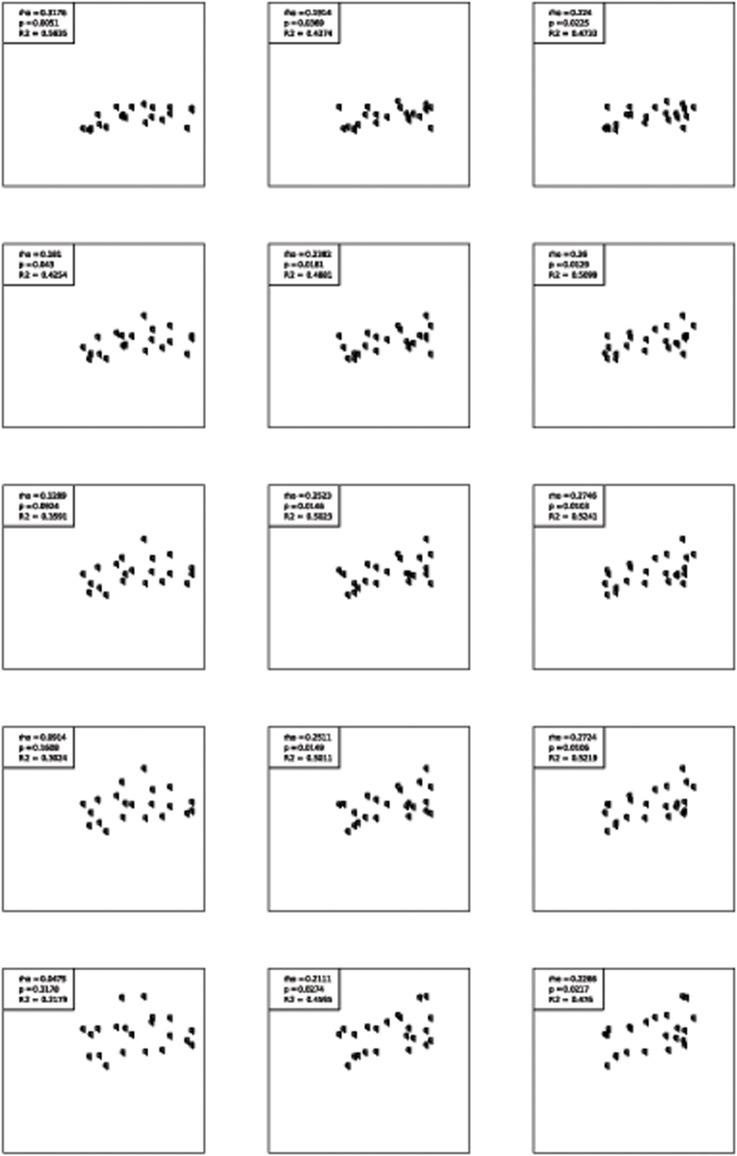
Association of 10mWT, 2MWT and 6MWT with different quantiles of real life walking speed. Walking speeds are based on sequences of at least 15 seconds uninterrupted walking, 6MWT = 6 Minute Walking Test, 2MWT = 2 Minute Walking Test, 10mWT = 10 meter Walking Test.

The results of the correlations between 10mWT and accelerometry data are shown in Table 3. Only lower quantiles (10 and 30%) of absolute walking speed, the mean number of steps per hour and walking speed in sequences with at least 50 steps were significantly correlated with the 10mWT walking speed. The best correlation was found for walking speed in sequences with at least 50 steps (R^2^ = 0.61, p < 0.01). Dotplots for different quantiles are presented in Fig 2. All other significant models had a low to moderate coefficient of determination R² (0.20–0.56) and the linear models explained only a small amount of the variability.

**Table 3:** Correlation between clinical gait test and daily life mobility parameters.

	6MWT (n = 23)		2MWT (n = 23)		10mWT n = (23)	
	p-value	R^2^	p-value	R^2^	p-value	R^2^
Quantiles of walking speed						
10%	0.02	0.47	0.04	0.44	0.01	0.56
30%	0.01	0.51	0.02	0.49	0.04	0.43
50%	0.01	0.52	0.01	0.50	0.09	0.36
70%	0.01	0.52	0.01	0.50	0.16	0.30
90%	0.02	0.48	0.03	0.46	0.32	0.22
Mean distance / day	0.47	0.17	0.08	0.38	0.88	0.03
Mean number of steps / hour	0.19	0.28	<0.01	0.69	0.01	0.50
Mean walking speed in sequence with at least 50 steps	<0.01	0.68	<0.01	0.79	<0.01	0.61
Mean walking speed in sequence with at least 100 steps	<0.01	0.71	<0.01	0.71	0.09	0.41

Walking speeds are based on sequences of at least 15 seconds uninterrupted walking, quantiles of walking speeds refer to the range of individual walking speeds with 0% the lowest measurable and 100% the fastest measurable walking speed, 6MWT = 6 Minute Walking Test, 2MWT = 2 Minute Walking Test, 10mWT = 10 meter Walking Test, R^2^ = coefficient of determination.

### 6MWT

Correlations between 6MWT and accelerometry parameters are included in Table 3. Most accelerometry walking speed parameters were significantly correlated with 6MWT, while mean distance per hour and mean number of steps per hour was not. The correlation of 6MWT with different walking speeds was consistently moderate (R^2^: 0.47–0.52). (Fig 2) The best correlations were found for sequences with at least 50 or 100 steps (R^2^ = 0.68 respectively 0.71).

### 2MWT

The results of the correlations between 2MWT and accelerometry parameters are as well summarized in Table 3. All but one accelerometry walking speed parameter were significantly correlated with 2MWT. Only mean distance per day was not. 6MWT, 2MWT correlated moderately with all real-life walking speeds. (Fig 2) The best correlation was found for sequences with at least 50 steps (R2 = 0.79). Results of the 2MWT and 6MWT were therefore comparable.

## Discussion

This study explored the ecological validity of standard clinical mobility tests in MS patients with a serial assessment of real-life mobility. Sequences of 6 minutes uninterrupted walking could hardly be detected in a representative mildly disabled MS cohort, implicating that the 6MWT does not represent a typical daily-life walking task. Even sequences of 2 minutes occurred only three times a day. The most frequent uninterrupted walking sequences in our cohort were not much longer than 15 seconds. These findings indicate that even if accelerometry data are assessed in real life the method itself must be studied in detail to guarantee that extracted data are meaningful and ecologic valid. Further investigations need to clarify which accelerometry data show the best association with patient relevant outcomes as quality of life, loss of productivity, need for care and mortality.

We used walking speed as primary outcome from the 7-day measurement as it is known that there is a continuous and robust decline of real life accelerometry gait speed with age and a lower variability compared to distance per day or number of steps.[16] Total movement counts were not included in this study as they are an established measure of physical activity rather than a specific mobility outcome.[29,30] Even if walking abilities contribute to total movement counts, motivational or behavioural factors have a major impact on this measures. Our data set did not allow a distinguished analysis of these factors. For the first time, we analysed precisely the association between different walking speed levels and clinical gait tests. The 10mWT showed only a moderate correlation with lower daily-life walking speeds, while speeds above the 30%-quantile of walking speed were not significantly associated. This contrasts the observation that walking speed during the clinical tests is about 1.4 m/s what represents the 75%-quantile of absolute daily-life walking speeds in our cohort, but are in line with the small analysis (n = 10) in traumatic brain injury patients by Moseley et al.[31] They compared 6MWT and 10m-walks with gait speed assessments in three real life situations (car park, shopping centre and hospital corridor) and observed a higher gait speed in the standard clinical tests.[31] Moseley suggested that these differences might rely on the “closed task” of clinical tests. A clear start and stop or the given time frame might represent a high motivational input to walk faster than usual.

Overall, 2MWT and 6MWT had a better and consistent correlation with all quantiles of real life walking speeds than the 10mWT but still explain only about a half of the variance of daily life walking speed. This moderate association confirms previous findings from Gijbels et al, who found similar correlations for habitual walking steps and 2/6MWT (R2: 0.42 and 0.46) while T25FW was even less predictive than the EDSS (R2: 0.31 and 0.33).[21] The findings from Motl et al. support as well a better correlation of accelerometry data with 6MWT than with T25FW.[22] The high association measured by spearman’s Rho in their study for the 6MWT (Rho: 0.63) with total movement counts might point towards the capability of the 6MWT to be more a measure of physical activity than of pure walking, which is as well closer to its original development as a measure of exercise capacity in cardiac failure. [32] Our analyses did not reveal major differences between 2MWT and 6MWT. This supports the suggestion, that the 2MWT might be seen as an alternative to the 6MWT.[8]

Due to the low to moderate correlation with real-life walking speed, the ecological validity of all performance based gait tests as generalizable measures for mobility is restricted but seems much better for 2MWT and 6MWT than for 10mWT. A precise estimate of real life walking speed seems a very robust putative outcome as it has been shown, that gait speed decreases with age and is inversely associated with quality of life and health status.[17,23,26,27] This is in line with previous studies that found a moderate but robust association between disability, 6MWT and real-life mean walking speed.[8,9,20,24,27,33] Short clinical tests might be ecologic valid measures of physical activity, while accelerometry represents mobility in daily life. This finding challenges the recommendation for short clinical gait tests like the T25FW as valid mobility outcome in MS.[9,10,34] An extensive analysis of T25FW data from the two dalfampidrine trials revealed that a 20% increase in speed in this test is clinically meaningful based on changes in the MSWS-12 self-reported walking scale.[34] In addition, benchmarks for disability levels, restriction in activities of daily living as well as the need for government support was associated with benchmarks of the T25FW.[35] However, this applies to more disabled patients and may not hold through in other cohorts.

We used mobile accelerometry as reference for ecological valid mobility assessments even though this approach has certain restrictions. Calculation of real-life walking speeds failed in more severely disabled patients. Therefore our findings are not transferable to patients needing a walking aid. Patients are still aware of “being measured”—at least initially—and the belt cannot be worn completely unseen with usual slim fit clothes. Further on, we were not able to compare the frequencies of uninterrupted walking periods with healthy individuals. Although, walking speed has been proven as robust marker for mobility with substantial correlation to strong outcomes such as morbidity or mortality, its relationship with as e.g. complex social behaviour has not been studied in depth.[5,7,36] As a limitation, we did not investigate the association between accelerometry data with self-assessment scales as the MSWS-12.[2] Ideally, serial self-assessment e.g. on a smart phone should be combined with a parallel real-life mobility assessment to gain further insight to the ecologic validity of self-assessment scales in MS. Accelerometry might be used in such a setting as a feedback mechanism to enhance physical activity.

Further studies are needed to determine the value of various potential optimizations of the technology platform using mobile accelerometry. For example, single real-life accelerometry parameters must be evaluated concerning their importance for social function, health and quality of life. However, the mobile accelerometry devices we have used in our study and similar devices allow now for the first time a statistically and theoretically meaningful, efficient and objective estimation of what is happening in real life. Motivational factors, personal background (e.g. profession) or time of year as potential covariates of clinical test performance and mobile accelerometry must be further examined in larger and prospective cohorts and its sensitivity to short and long-term changes needs further investigations. Mobile accelerometry might be promising outcome and monitoring tool in MS studies as well as in other neurological diseases. Chabrol and Harrer have recently highlighted this.[37]

## Conclusion

2MWT and 6MWT showed a moderate and similar correlation pattern with real-life walking speeds but uninterrupted 2- or 6-minutes walks are uncommon in real life. The 10mWT correlated only with lower quantiles of real life walking speeds and has poor ecologic validity while 2MWT and 6MWT correlated moderately. Mobile accelerometry offers the opportunity to control and improve the ecological validity of MS mobility outcomes.
